# Current hurdles to the translation of nanomedicines from bench to the clinic

**DOI:** 10.1007/s13346-021-01024-2

**Published:** 2021-07-23

**Authors:** Snežana Đorđević, María Medel Gonzalez, Inmaculada Conejos-Sánchez, Barbara Carreira, Sabina Pozzi, Rita C. Acúrcio, Ronit Satchi-Fainaro, Helena F. Florindo, María J. Vicent

**Affiliations:** 1 Polymer Therapeutics Laboratory, Prince Felipe Research Center (CIPF), Eduardo Primo Yúfera 3, 46012 Valencia, Av Spain; 2 Research Institute for Medicines (iMed.ULisboa), Faculty of Pharmacy, Universidade de Lisboa, Avenida Professor Gama Pinto, 1649-003 Lisboa, Portugal; 3 Department of Physiology and Pharmacology, Sackler Faculty of Medicine, Tel Aviv University, 69978 Tel Aviv, Israel; 4 Sagol School of Neuroscience, Tel Aviv University, 69978 Tel Aviv, Israel

**Keywords:** Nanomedicine translation, Regulatory framework, Manufacturing, Scale-up, Characterization

## Abstract

The field of nanomedicine has significantly influenced research areas such as drug delivery, diagnostics, theranostics, and regenerative medicine; however, the further development of this field will face significant challenges at the regulatory level if related guidance remains unclear and unconsolidated. This review describes those features and pathways crucial to the clinical translation of nanomedicine and highlights considerations for early-stage product development. These include identifying those critical quality attributes of the drug product essential for activity and safety, appropriate analytical methods (physical, chemical, biological) for characterization, important process parameters, and adequate pre-clinical models. Additional concerns include the evaluation of batch-to-batch consistency and considerations regarding scaling up that will ensure a successful reproducible manufacturing process. Furthermore, we advise close collaboration with regulatory agencies from the early stages of development to assure an aligned position to accelerate the development of future nanomedicines.

**Graphical abstract:**

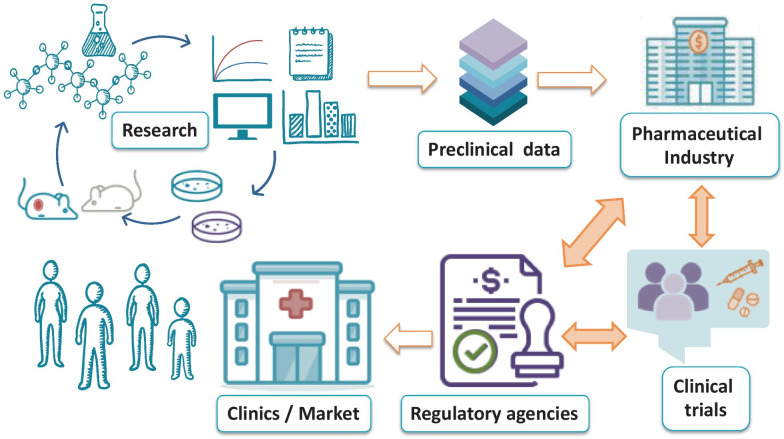

## Introduction

Nanotechnology represents a vital resource in a range of distinct but related fields that include engineering, chemistry, physics, and biotechnology; however, nanotechnology represents the “shining star” of the health sciences and has induced a paradigm shift in the way the scientific community addresses unmet medical needs. Currently marketed nanomedicines have a broad range of clinical applications in the prevention, diagnosis, and treatment of various diseases [1]. Advanced nanotechnology-based strategies aim to overcome the limitations of free drugs and promote passage through biological barriers that function as impediments to therapeutic agents. Targeted and non-targeted nanomedicines can improve the stability and solubility of associated active payloads, promote transport across membranes, and prolong circulation times to increase safety and efficacy [2, 3]. There currently exists around 100 nanomedicines approved by various regulatory agencies worldwide (Table 1), with others under advanced pre-clinical and clinical evaluation [4–7].

**Table 1:** FDA- and EMA-approved nanomedicines since 2015, updated from Sainz et al. [13]

Tradename	Nanoplatform and active agent	Application	Approval (date)	Company
Adynovate®/Adynovi®	PEGylated recombinant anti-hemophilic factor	Hemophilia	FDA (2015) EMA (2017)	Takeda
Aristada®	Aripiprazole lauroxil nanocrystals	Schizophrenia	FDA (2015)	Alkermes
Glatopa® (Generic of Copaxone)	Random copolymer of L-glutamate, L-alanine, L-lysine, and L-tyrosine	Multiple sclerosis	FDA (2015)	Novartis
Invega Trinza®/Trevicta®	Paliperidone palmitate nanocrystals	Schizophrenia	FDA (2015) EMA (2016)	Janssen
Inveltys®	Loteprednol etabonate nanosuspension	Post-operative ophthalmic inflammation	FDA (2018)	KALA pharmaceuticals
Mircera®	PEGylated epoetin beta	Anemia in chronic renal diseases for pediatric patients	FDA (2018) EMA (2019)	Vifor
mRNA-1273	Lipid nanoparticle of full-length, prefusion stabilized spike protein mRNA	Prevention of COVID-19	FDA (2020) EMA (2021)	Moderna
Onpattro® (Patisiran)	Lipid nanoparticle for siRNA targeting TTR protein	TTR-mediated amyloidosis	FDA and EMA (2018)	Alnylam Pharmaceuticals
Onivyde®	PEGylated liposomal irinotecan	Metastatic pancreatic cancer	FDA (2015) EMA (2016)	Merrimack
Rebinyn®/Refixia®	PEGylated glyco-protein drug	Hemophilia	FDA and EMA (2017)	NovoNordisk
Sublocade®	Buprenorphine-loaded PLGA nanoparticles	Opioid use disorder	FDA (2017)	Indivior
Tozinameran®	Lipid nanoparticle of full spike mRNA	Prevention of COVID-19	FDA and EMA (2020)	BioNTech SE and Pfizer
Vyxeos®	Liposomal formulation of cytarabine: daunorubicin (5:1 M ratio)	Acute myeloid leukemia	FDA (2017) EMA (2018)	Jazz Pharmaceuticals
Zilretta®	PLGA hydrogel of triamcinolone acetonide	Knee osteoarthritis	FDA (2017)	Flexion Therapeutics

*FDA* Food and Drugs Administration, *EMA* European Medicines Agency, *TTR* transthyretin, *PLGA* poly(lactic-co-glycolic acid), *PEG* polyethylene glycol, *COVID-19* coronavirus disease 2019

The novel coronavirus disease 2019 (COVID-19) pandemic has fostered significant interest in the application of nanomedicine to global healthcare problems; 1 year after the first case of severe acute respiratory syndrome coronavirus 2 (SARS-CoV-2) infection, two mRNA nanomedicine vaccines received emergency use authorization from the US Food and Drug Administration (FDA) and European Medicines Agency (EMA), while others remain under clinical evaluation [8, 9]. No other oligonucleotide-based nanomedicine has been developed and authorized at the same pace, considering, for example, the amyloidosis therapeutic Onpattro® (Patisiran) [10]. This RNA interference-based lipoplex for the treatment of transthyretin-mediated amyloidosis was designated as an orphan medicinal product (EU/3/11/857) in April 2011 and approved in August 2018, a considerably different time frame compared to mRNA vaccines [11, 12] (Table 1). The scientific achievements made in response to the COVID-19 pandemic have streamlined years or even decades of challenges and hurdles in the clinical translational pathway faced by nanomedicines. Indeed, fast-tracking and rapid approval, which maintain the highest standards for safety evaluation but require less bureaucracy, have provided hope for the future of nanotechnology-based products.

While nanomedicine represents a rapidly growing market that combines benefits, risks, and promises, it suffers from a degree of uncertainty. Recent events have significantly impacted the nanomedicine market and perhaps eased approval mechanisms in others; however, nanomedicines still face significant challenges as the regulatory guidance remains unclear and unconsolidated. Of note, the 2017 non-binding draft guidance document from the FDA for drug products containing nanomaterials prompted some progress; however, this did not obviate the need for more robust guidelines. The consolidation of general protocols concerning the pre-clinical development and physico-chemical characterization steps required to translate nanomedicine into the clinic represents an important step in overcoming the reticence exhibited by numerous pharmaceutical companies to devote efforts towards nanomedicine development. The present review discusses the requirements at distinct levels, challenges, and opportunities concerning nanomedicine translation.

### Current regulatory approaches for nanomedicines

Although many nanotechnology-based products have received approval for medical applications (Table 1), worldwide regulatory ethics and policies remain unestablished or undeveloped, thereby hindering the potential of nanomedicines.

Among the hurdles associated with nanomedicine regulation (Box 1), the main challenges relate to the inherent properties of nanomedicines. These include distinct pharmacodynamic (PD) and pharmacokinetic (PK) profiles compared to their associated constituent materials and payloads [14].

Current regulations addressing the safety and efficacy of standardized small drug molecules should be considered when evaluating nanomedicines. Furthermore, the impact of physicochemical properties on the biodistribution and interaction of nanotechnology-based products with tissues and biological membranes should be considered by regulators [15]. The definition and classification of nanotechnology-based products represent additional challenges—while related products can be classified as medicines or medical devices, a lack of consensus exists across the globe. For this reason, the regulatory framework for a given nanomedicine will change according to the country, thereby hindering approval and regulation [16].

While questions remain concerning the entity/entities that should determine global nanomedicine guidelines, international regulatory agencies have convened specialized multidisciplinary groups composed of academics, clinicians, and regulators to work towards a universal objective of drafting agreed worldwide guidelines for evaluating and regulating nanotechnology-based products.

**Box 1** Summary of the major challenges associated with nanomedicine regulation **Challenges hampering nanomedicine regulation**

• Lack of a unified definition or classification of nanomedicines/nanomaterials [17–19]

• Lack of agreed regulations [17–19]

• Analytical methods differ for each nanomaterial [13, 20, 21]

• PK profiles diverge from standardized constituent materials [22, 23]

• Stability issues after scale-up for manufacturing [13, 21, 24, 25]

• Current in vitro and pre-clinical toxicological studies fail to mimic in vivo complexity [26–31]

• Systemic biodistribution and fate [3, 31–34]

• Possible environmental impact [17, 19, 30]

### The European Union and the United Kingdom

Within the European Union (EU), the EMA applies the *General Medicinal Product* legislation to nanomedicines, with a legal reference regarding nanomaterials published in 2011 (Recommendation 2011/696/EU) by the European Commission (EC) [35]. This recommendation sets the first suitable definition of “nanomaterial” within the EU for legislative and policy use; however, this recommendation is not legally binding nor imposed across the EU. The European Medicines Union (EMU), a regulatory body of the EU, has begun to issue specific preliminary guidelines to standardize nanomedicine preparation standards; however, official regulatory guidelines remain unpublished [36–38]. Task forces and consortiums, including the *Nanomedicines Expert Group* (formed by the EMA), have sought to establish different initiatives, which include the Nanomedicine Characterization Laboratory (NCL) and the Regulatory Science Framework for Nano(bio)material-based Medical Products and Devices (REFINE) project (http://refine-nanomed.eu/), to form definitions and guidelines for the regulation of nanomedicines and provide constantly updated knowledge on pre-clinical characterization methods [39–41]. The EU currently regulates nanomedicines using risk/benefit-analysis principles. The EU-funded European Chemical Agency (ECHA) addresses the safety of chemicals (including nanomaterials) under the regulation of the European Chemical Legislation (REACH EC 1907/2006) [42]. The amendment of the REACH legislation in 2018 introduced new requirements for chemical safety assessment of nanomaterials and obligations for downstream users [43]. Another EU regulatory agency, the European Food Safety Authority (EFSA), takes responsibility for risk assessments of nanomaterials within foodstuffs for human and animal consumption. The EFSA has published beneficial guidance for risk assessments and uncertainty analysis for nanomaterials in foodstuffs and has provided recommendations for further research [44, 45].

In the United Kingdom (UK), the Medicines and Healthcare Products Regulatory Authority (MHRA) regulates all medicines, thereby encompassing nanomedicines. As in the EU, the UK lacks specific guidelines for nanotechnology-based products. The approval of nanomedicines by the MHRA is managed on a case-by-case basis, with researchers encouraged to communicate with the MHRA for support throughout the development process.

### The United States of America

While the United States of America (USA) also lacks specific nanomedicine guidelines, the FDA has published regulatory frameworks for nanomedicines in foodstuffs [46], cosmetics [47], and animal feed [48]. The FDA regulates nanotechnology-based products on a case-by-case basis using statutory and regulatory authorities with product-specific standards [49]. The FDA first published draft guidance on drug products in 2017, including biological products containing nanomaterials [46]. As in the UK, the FDA encourages their consultation during the development process of any nanotechnology-based products regarding safety information, regulatory issues, and marketing. After approval, the FDA continues to monitor nanomedicines to protect consumers and advise manufacturers about safety, given their responsibility for ensuring nanomedicine obedience to legal requirements. The Nanotechnology Characterization Laboratory of the National Cancer Institute (NCL-NCI) also contributes to nanomedicine regulation [50, 51]. Close collaborations between the FDA and the US government departments and agencies through the *National Nanotechnology Initiative* (NNI) aims for early dialogue during product development. The NNI focuses on preparing guidance documents for the characterization and quantification of nanomaterials based on six areas: measurement infrastructure, human exposure assessment, human health, environment, risk assessment and management, and informatics and modeling [51].

The FDA also established the *Nanotechnology Task Force and Nanotechnology Interest Group* to meet demanding issues in nanomedicine regulation; however, no specific guidelines have been issued, and already established guidelines have been accepted as sufficient. Based on this assumption, nanomedicines continue to be evaluated and regulated as for other drugs/therapeutics, with the belief that established guidelines will uncover potential problems and, therefore, guide safety decisions. Therefore, nanomedicines prepared using existing approved components move rapidly through regulatory procedures as no additional pharmacotoxicology studies would be needed to address the safety of the individual parts to those required for the nanomedicine as a whole new chemical entity (NCE). The absence of changes in nanomedicine regulation guidelines has raised concerns and evoked criticisms of the FDA; however, the establishment of general industry guidelines related to liposomal-drug products represents an important step towards the construction of regulatory frameworks for nanomaterials [52] and could prompt the establishment of draft guidance for other types of nanomedicines. The FDA is constantly reviewing approval submissions for nanomaterial-containing products, with many in clinical trials and others approved for applications in medical devices and future drugs [51, 53].

### Canada

Canada regulates nanotechnology-based products based on existing guidelines recommended by the Organization for Economic Co-operation and Development (OECD) Council for the safety assessment of nanomaterials [54, 55]. As for the EU and USA, Health Canada supports and advises manufacturers during the development process by assessing the risks and properties of nanomedicines [56]. The establishment of the Canadian Health Portfolio Nanotechnology Working Group aimed to support regulatory agencies (Health Canada and the Canadian Institutes of Health Research) to discuss and categorize nanotechnology-based concerns. To date, Health Canada has published draft guidance for nanotechnology-based health products and foodstuffs [57].

### Asia

India, Japan, China, and Thailand are currently creating regulatory guidelines relevant to nanomedicines. In India, the government and the Department of Science and Technology have established a working group responsible for regulating and drafting guidelines for nanotechnology-based products. In 2019, the Indian government published the first guidelines for nanomedicine regulation, covering the development of new drugs and their comparison with existing entities [51, 58]. In Japan, the Ministry of Health, Labor and Welfare (MHLW) and the Pharmaceuticals and Medical Devices Agency are responsible for this process [59], publishing specific guidelines for the regulation of liposome-based drug products in 2016. As in the USA and EU, the Pharmaceutical Affairs Law framework in Japan legislates nanomedicines on a case-by-case basis in close collaboration with the EMA.

### International

International pharmaceutical regulation is the responsibility of the International Pharmaceutical Regulators Program (IPRP) under the scope of the International Council for Harmonization of Technical Requirements for Pharmaceuticals for Human Use (ICH). The IPRP comprises a working group that includes America, Asia, Europe, and Oceania and covers emerging issues related to nanomedicines and nanomaterials in drug products. Their main objective is to establish harmonized regulatory frameworks for nanomedicines—namely, what information needs to be reported to regulators—by maintaining close collaborations with all international regulatory agencies [51]. The previously noted lack of specific guidelines for the adequate characterization of nanomedicines at the physicochemical and physiological levels may have contributed to the failures of certain nanomedicines at late clinical stages [13, 60]. Reflection articles currently provide limited guidelines on the pharmaceutical development of specific nanomedicines [61]; however, defining the parameters that must be considered to adequately evaluate nanomedicine quality control and safety and associating those parameters to a regulatory definition by differentiating active pharmaceutical ingredients (APIs), excipients, and drug products from a physico-chemical point of view remain important tasks.

### Parameters to consider for quality and safety evaluations of nanomedicines

#### Active pharmaceutical ingredients and excipients

An API is defined in the ICH quality guideline (Q7) as “any substance or mixture of substances intended to be used in the manufacture of a drug product and that, when used in producing a drug, becomes an active ingredient in the drug product. Such substances are intended to furnish pharmacological activity or carry out other direct effects in the diagnosis, cure, mitigation, treatment or prevention of disease or to affect the structure and function of the body” [62]. ICH Q7 contains industrial quality guidelines on the extent and application of good manufacturing practice (GMP) for APIs under an appropriate management quality system [62]. The quality requirements of a given API or active substance aid the manufacturer in determining ingredient use and permissible usage limit. The consideration of a nanomedicine as an API depends on sponsor regulatory strategy, in addition to the method employed for drug association (conjugation or encapsulation). Few nanomedicines are classed as APIs, being rather mostly classified as prodrugs. The safety of the individual components of complex therapeutics must be proved unless they have been in clinical use for many years.

APIs represented a 121 billion USD market in 2016, which will reach nearly 200 billion by 2022 [63]. The rapid evolution and challenges posed by new classes of therapies in the pharmaceutical industry have prompted the emergence of new nomenclature that distinguishes generic small molecules and biological drugs from non-biological drugs. The non-biological complex drug (NBCD) working group, an initiative hosted by the Top Institute Pharma (Leiden, The Netherlands), defines NBCDs as a non-biological medicinal product where APIs consist of closely related (and often nanosized) structures that, instead of being considered as separate moieties, are characterized and described as one whole entity. Examples of NBCDs include iron-carbohydrate complexes, glatiramoids, liposomes, polymeric micelles, and swelling polymers [64, 65]. The regulation of biosimilars (defined as a biologic medical product highly similar to an already-approved biological medicine) has paved the way for NBCDs, and, therefore, similar criteria will likely be adopted for the approval of “nanosimilars” [21, 64, 65].

Fully appreciating the quality requirements for APIs requires an understanding of the three main aspects of a given API—their chemical, physical, and biological properties.

1. Chemical properties: All chemical reactions involve the generation of impurities from active substances or solvents. Impurities are mainly formed during synthesis, where the product becomes contaminated by raw materials, solvents, intermediates, and by-products. Such impurities are classified by ICH Q3A Guidelines [66] into:

- Organic impurities (process- and drug-related): Such impurities can be identified or unidentified, volatile, or non-volatile, and include starting materials or intermediate products (the most common impurities found in any API unless proper care are taken in every step involved in the synthesis), reagents, and components implicated in the reaction, such as catalysts (e.g., copper derivatives), and enantiomeric impurities. Degradation products of the drug substance or reaction products deriving from the interaction of the drug substance with an excipient and/or immediate container closure system deserve special consideration. Generally, impurities present in a new drug product must be monitored and specified according to the ICH Q3B guideline [67].
- Inorganic/elemental impurities: Elemental impurities must be monitored to ensure their levels remain below the acceptable limit. ICH Q3D Guidelines classify elemental impurities into different classes that focus on risk assessments of the most toxic elements and those with a reasonable probability of inclusion in the final drug product. Heavy metals, the most common elemental impurities, can be avoided using demineralized water and glass-lined reactors during synthesis.
- Residual solvents: Manufacturers must consider the potency of ingredients and solvents used during production and control levels to avoid toxicities [68]. The most common residual solvents associated with nanomedicine production are chloroform (EMA concentration limit—60 parts-per-million [ppm]) or N,N-dimethylformamide (880 ppm), which can be detected by techniques such as ^1^H-NMR or mass spectrometry (MS).

(2) Physical properties: The particle size, proportion, and polymorphism of an API may affect physical properties, rates of absorption, and bioavailability and represent critical factors that determine drug use. Carefully controlling physical properties can ensure the solubilization of an API in body fluids.
(3) Biological properties: The manufacturer must ensure control over bioburden (i.e., the presence of microbes). Sterility and endotoxin levels should remain under levels stated in Regulatory Agency guidelines [69]—a critical point for all administration routes but especially for parenteral administration [70]. NCL assay protocols STE-1, -2, -3, and -4 provide procedures for sterility assurance and endotoxin quantification. In addition, the ICH M7(R1) guideline describes assays for the identification, categorization, qualification, and control of DNA reactive impurities to limit potential carcinogenic risk [71].

Few APIs possess all properties required for consideration as a final drug product and require further formulation [72]. The FDA defines “any component of a pharmaceutical product other than the API” as an excipient [73], which are implemented in nanomedicine production as anti-aggregation agents during processing, off-the-shelf stability agents, solubilizing agents, hydrophilization agents, or viscosity enhancers, among others. Excipients also provide additional pharmacological properties/functions, including mucoadhesion [74], enzyme inhibition [75–77], oral absorption enhancement [78], efflux pump inhibition [79], or taste-masking [80].

In solid particle excipients (and not a molecule or suspension medium), the chemical composition, size, surface charge, and morphology of said particle can affect the therapeutic agents’ properties, such as product performance, processability, stability, toxicity, and appearance. In this regard, the characterization of the nanosized excipient remains as important as the characterization of the API.

### Drug product

#### Manufacturing and scale-up

The most challenging steps in nanomedicine product development derive from the transition from small laboratory batch size to large industrial volumes and the selection of excipients required to produce high-quality pharmaceuticals [81]. Research and development methods often involve low-volume production, and scaling can pose severe challenges to producing specific nanomaterials [82].

The manufacturing of a nanomedicine product involves a multi-step process that requires the constant control of the nanomaterial properties (size, shape, charge, structure, composition, physicochemical, PK, and biopharmaceutical properties [82, 83]). The development of nanomedicines relies on progress made in manufacturing technology to accommodate scalable processes complying with GMP quality guidelines. The application of GMP ensures the quality of processes and obtained products, requiring detailed written procedures for each process that affect the finished product’s quality [84]. Moreover, the application of GMP guidelines minimizes the risks involved in all aspects of production, from the starting materials and equipment to training personal. GMP systems also provide documented proof of the implementation of correct procedures at each step of the manufacturing process.

In 2005, the pharmaceutical industry worked with the EC to set up The European Technology Platform on Nanomedicine (ETPN), an initiative to address the application of nanotechnology in healthcare. The ETPN aims to create conditions for the successful translation of nanomedicines by shaping and supporting public funding in promising areas of nanomedicine research and designing a unique technical infrastructure—the Nanomedicine Translation Hub [85]. The ETPN Nanomedicine Translation HUB offers custom mentoring through Translation Advisory Boards, product characterization through the NCL, and GMP manufacturing through pilot lines. These free-of-charge services are open to all, including entrepreneurs, SMEs (small- and medium-sized enterprises), industry, and academic labs, and aim to remove specific roadblocks to nanomedicine product development and support/accelerate clinical development of promising nanomedicine research [86, 87]

A wide range of nanomaterial-specific production methods for nanomedicines has been reported. Table 2 represents a summary of the production methods associated with nanoparticles with the potential to reach clinical trials and the critical factors that determine their choice [88].

**Table 2:** Examples of the most used methods to produce nanomedicine

	Lipid-based nanomedicines	Inorganic/metal-based nanomedicines	Polymeric-based nanomedicines
Methods of production	High-pressure homogenization (hot and cold) Membrane contractor method Microemulsion Solvent diffusion Solvent evaporation Ultrasound High-shear homogenization	Chemical methods (metal complex reduction) Physical methods (laser pulses, supercritical fluid, chemical vapor deposition, microwave radiation)	Extrusion Ionic gelation Nanoprecipitation Salting out Supercritical fluid Bioconjugation Tangential flow filtration
Critical factors	Hydrophilicity of drug Polydispersity index Particle size Lab-scale vs. Industrial-scale Temperature Organic solvent Structural organization	Hydrophilicity of drug Polydispersity index Particle size Type of solvent Organic solvent Surface-to-volume ratio	Polydispersity index Particle size Non-volatile impurities as bioconjugation subproducts Structural organization Bioresponsiveness

**Fig. 1:**
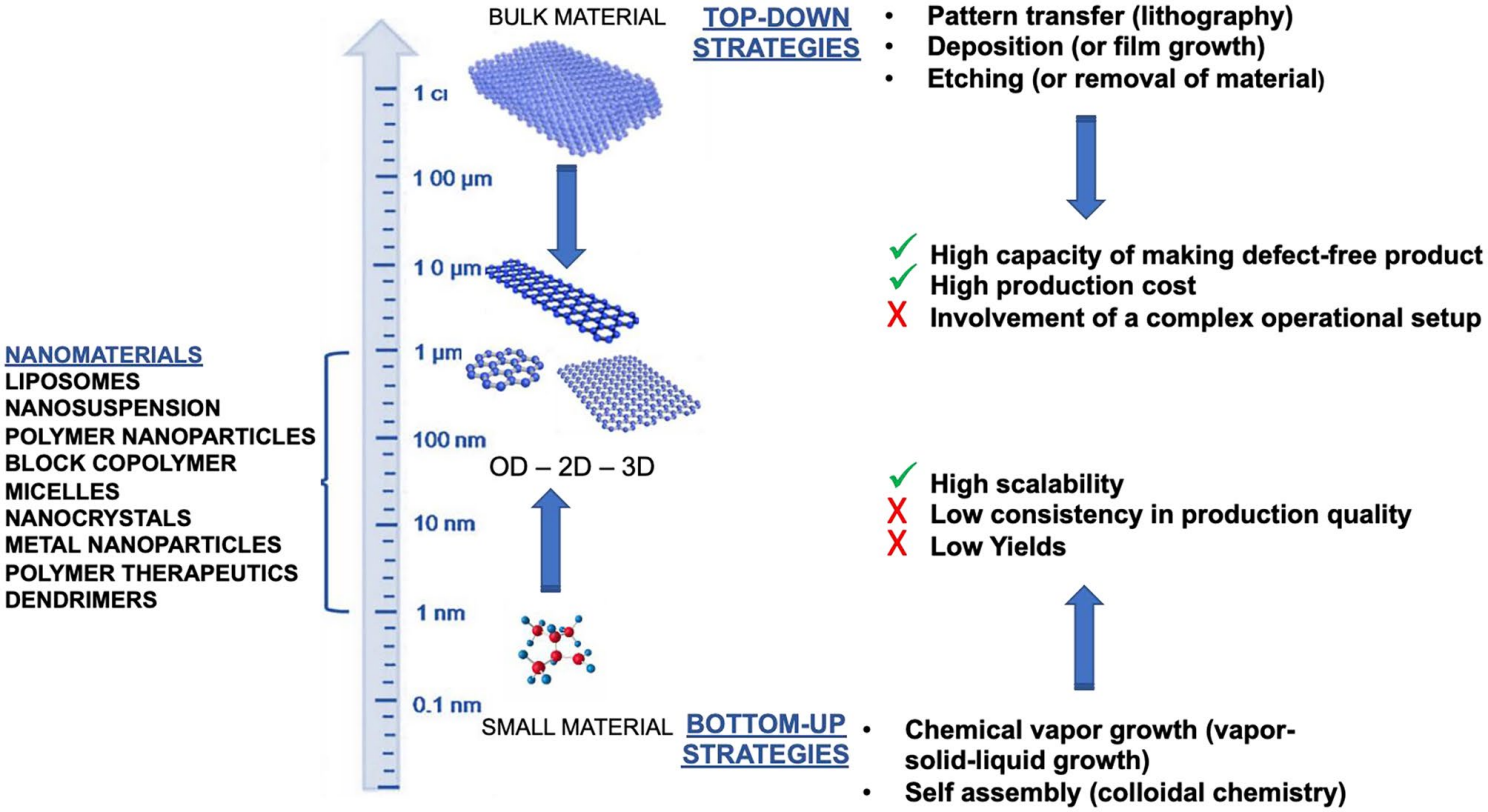
Approaches for nanomedicine manufacture

The manufacturing of nanomedicines generally uses one of two approaches, either “top-down” or “bottom-up” (Fig. 1) [89–91].

Top-down approaches employ larger (macroscopic) starting structures that can be externally controlled during processing and transformed into nanostructures via the application of severe plastic deformation using mechanical, chemical, or other forms of energy. Examples include wet media milling or bead milling (shear forces), the production of emulsions in high‐pressure homogenizers (HPH) (cavitation force), grinding, and extrusion. Such approaches have supported the approval of several nanocrystal formulations (e.g., Tricor, Triglide, Emend, Rapamune, Megace ES, Invega sustena, and Ampyra) [88, 92].

Top-down fabrication methods easily control particle size and shape and permit an extensive examination of the versatility of particle replication in non-wetting templates (PRINT) technology [93], which can tailor physico-chemical parameters of nanoparticles for later exploration of single formulation variables and particle biodistribution [93]. Studies demonstrated that decreased particle diameter reduced liver and spleen accumulation and improved tumor accumulation [94].

Bottom-up approaches refer to constructing a complex material from simpler constituents—atom-by-atom, molecule-by-molecule, or cluster-by-cluster. While displaying greater versatility and yielding complex nanomedicines, the bottom-up approach remains challenging for industrial implementation and presents problems to scale-up efforts. Said problems suggest the implementation of quality-oriented product manufacturing [95]. Examples of bottom-up approaches include sol–gel processing, precipitation, aerosol-based, chemical vapor deposition (CVD), plasma or flame spraying synthesis, laser pyrolysis, atomic or molecular condensation, microfluidics, nanoemulsions, and chemical conjugation and crosslinking, among others. The manufacture of liposomal formulations (including Doxil® [95]) follows bottom-up approaches [96]. Polymeric nanoparticles at the laboratory scale can be obtained by solvent evaporation, salting-out, emulsification-diffusion [97], or solvent displacement, among others; however, the latter has been associated with poor industrial-scale translation [82]. Alternatively, supercritical precipitation, electrospraying, or spray-drying have seen more success at the production level. Self-assembly strategies [98, 99] and bioconjugation approaches, such as those required to achieve polymer-drug conjugates [100] or antibody–drug conjugates [101], present additional chemical and analytical challenges.

Both top-down and bottom-up approaches can be implemented in gas, liquid, supercritical fluid, solid states, or in a vacuum environment; however, the critical attributes under control remain similar.

### Requirements of quality management systems for next-generation nanomedicines

The control and adaption of manufacturing processes and production scale-up represent additional stumbling blocks encountered during the development and clinical translation of nanomedicines, primarily due to the extensive diversity of nanomedicines [65]. Implementing quality management systems (QMSs) can overcome these drawbacks and ensure that the final product fulfills the regulatory agencies' specifications. Thus, QMSs can significantly contribute to a given nanomedicine’s clinical success and operational excellence by identifying and controlling the critical points of each manufacturing process. QMS must be designed as a reference framework of a quality policy, fulfilling established standardized documents whose technical specifications and criteria guide the development and manufacturing phases of production; however, a formal, practical, systematic, and robust assessment of risks with validated quality protocols specific for nanomedicines is urgently required at the global level.

A risk-based concept known as Quality-by-Design (QbD), introduced by the FDA and ICH in 2000 and 2008, respectively, supports the identification, analysis, and control of factors critically impacting an NCE’s quality and safety. QbD represents a powerful tool that minimizes product defects, reduces waste and environmental risks, and positively impacts health and safety [102]. Unlike traditional approaches that only evaluate final product quality, QbD ensures and controls product quality and safety throughout the manufacturing process. Thus, QbD requires a thorough comprehension of the variabilities in the attributes of the raw materials involved, the relationship between a process and a product’s critical quality attributes (CQAs), and the association between CQAs and the product's clinical properties [103]. Generally, QbD is implemented in several steps: (i) the establishment of the Quality Target Product Profile (QTPP) for a nanosized product (name, dosage form, route of administration, clinical intended use, PK, etc.); (ii) identification of CQAs (physicochemical or biological properties to be controlled); (iii) identification of parameters that influence process performance; (iv) risk assessment analysis to identify risk parameters; (v) implementation of the Design of Experiment (DoE) approach to evaluate how critical parameters influence CQAs; (vi) a process design space definition that originates a final product with the desired QTPP; (vii) risk control strategy (RCS) development to identify causes of variability; and (viii) continuous monitoring and improvement of the manufacturing process, which forms a Product Life Cycle Management (PLM).

**Fig. 2:**
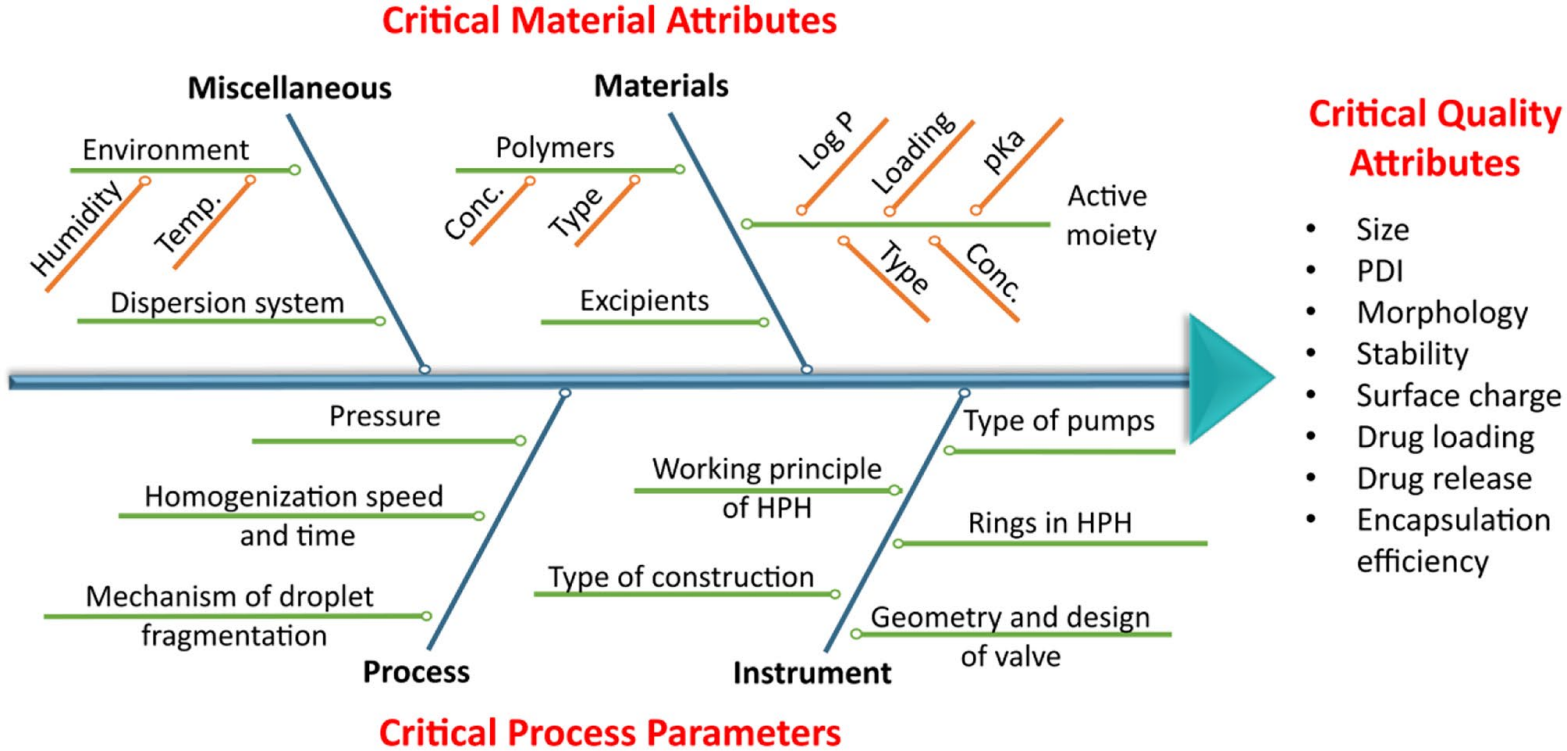
Representation of those critical material attributes (CMA) and critical process parameters (CPP) that cause discrepancy and variability of critical quality attributes (CQAs) during the rational design and production of nanomedicines

The accurate identification of CQAs remains a crucial step in the quality and safety assessment of nanomedicines under development. There exists a large variety of CQAs used in pre-clinical studies (Fig. 2); however, nanomedicine size, encapsulation efficiency, polydispersity index, zeta-potential, and drug release kinetics represent the most studied parameters [104]. To address the need for continuous analysis of CQAs, the FDA introduced changes in cGMP in 2002 and outlined the importance of Process Analytical Technology (PAT) in innovative pharmaceutical development, manufacturing, and quality assurance [105, 106]. PAT includes a real-time assessment of critical quality and performance attributes of processes and raw and in-process materials that could provide the basis for continuous feedback and result in improved process robustness [107, 108].

The crucial factors that cause the most significant discrepancy and variability of CQAs during the rational design and production of nanomedicines are considered critical material attributes (CMA), associated with the formulation parameters (i.e., ingredient concentration, ingredients ratio, drug load, and surfactant concentration), and critical process parameters (CPP), associated with nanomedicine production parameters [109] (Fig. 2). Many manufacturing processes do not allow the identification of dominant CPPs for nanomedicines; instead, the most crucial CPPs among evaluated formulation parameters can be specified for each nanomedicine subgroup (i.e., temperature and homogenization speed and time or energy processing time, amplitude, or pressure as CPPs for lipid nanoparticles and nanoemulsion formulation). Statistical tools such as risk assessment analysis [110] and DoE [111–114] allow the classification and estimation of the criticality of identified factors that may increase production efficiency and reduce manufacturing waste.

The definition of CQAs, CMPs, CPPs, and design space does not represent the ultimate goal of QbD; additionally, the last QbD steps (RCS and PLM) cannot be ignored. RCS includes in-process controls, finished product specifications, and the associated methods and frequency of monitoring and control. The implementation of adequate analytical methods at the correct time represents a crucial component of RCS. Applying QbD to analytical methods used in pre-clinical research remains a matter of debate; furthermore, whether one should invest time in this matter when clinical safety and efficacy remain unproven remains an open question. However, the careful quality control of synthesized nanomedicines with fit-for-purpose and robust analytical methodologies can improve reproducibility in both pre-clinical and clinical efficacy studies [100]. PLM processes include the management of a product’s entire lifecycle from establishment to disposal. Therefore, PLM represents an indispensable tool to compare QbD estimated risks with clinical results and continuously improve nanomedicine quality and safety.

QbD can provide numerous advantages to the pharmaceutical industry by ensuring the enhanced design of products with fewer manufacturing problems, allowing continuous manufacturing improvement, enabling reductions in manufacturing costs and waste, and most importantly, providing a better understanding of how APIs and excipients affect manufacturing and manufacturing processes related to the clinic [103]. However, few QbD steps have been applied to nanomedicine, primarily due to the relative complexity of QbD implementation and statistical analytic skills required. Moreover, the optimized identification of nanomedicine-specific CQAs represents an essential step [109]. Although the manufacturing processes for both small drugs and nanomedicines display similarities, the latter suffers from unique issues that must be addressed before QbD implementation.

### Analytical techniques for quality control manufacture

Before moving to clinical studies, all nanomedicine candidates must be characterized by robust, straightforward, and affordable methods to ensure a high-quality product and control the physico-chemical descriptors (and other factors) influencing efficacy and safety [115].

Compared to conventional small molecule drugs, the characterization of nanomedicines demands an assessment of subgroup-dependent physico-chemical properties that influence the quality, safety, and efficacy profiles. However, chemical composition, average particle size, size polydispersity, particle shape and morphology, and physical and chemical stability [116] represent common critical parameters that should be described for any nanomedicine according to regulatory agency guidelines. Furthermore, these properties should be evaluated in relevant biological media, where the presence of various organic molecules can modify a given nanomedicine and prompt degradation, agglomeration, or protein corona formation [117]. Depending on the nanomedicine type, administration route, and indication, additional parameters may also be of interest—these include surface properties (e.g., charge, hydrophobicity, surface area, and chemical reactivity), structural attributes (e.g., core–shell structure, coating, porosity, and crystallinity), particle concentration, impurities, endotoxin levels, total drug loading (TDL), free drug percentage compared to TDL, and drug release kinetics [116]. The variety of materials employed and the complexity of synthetic techniques involved require distinct methodological approaches to characterize the physico-chemical properties of nanomedicines.

The FDA and EMA evaluate drug product quality from a safety and efficacy perspective during drug approval. Once approved for human use, respective pharmacopeias (such as the US Pharmacopoeia [USP] and the European Pharmacopoeia [Ph. Eur]) develop a product monograph, which describes critical physicochemical aspects, specific analytic methods, and API formulation and include product specifications and the justification for those specifications. While the Ph. Eur. and USP provide standardized methods for quality assessment and characterization of approved APIs, neither provide nanomedicine-specific methods given that currently-approved nanomedicines generally remain under patent protection. The guidelines, standardized evaluation methods, perspectives, and reports dedicated to the physicochemical and biological characterization of engineered nanomaterials have been published by the EMA [118–120], FDA [52, 116], the International Organization for Standardization (ISO), and the American Society for Testing and Materials (ASTM International). An ISO standard (ISO/TR 18,196:2016) published in 2016 guides users to commercially available techniques for measuring standard physico-chemical parameters for nanosized objects and nanostructured materials [121]. Nevertheless, this ISO standard only represents a resource to identify practical, relevant techniques and does not represent a set of guidelines for nanomedicine characterization.

**Fig. 3:**
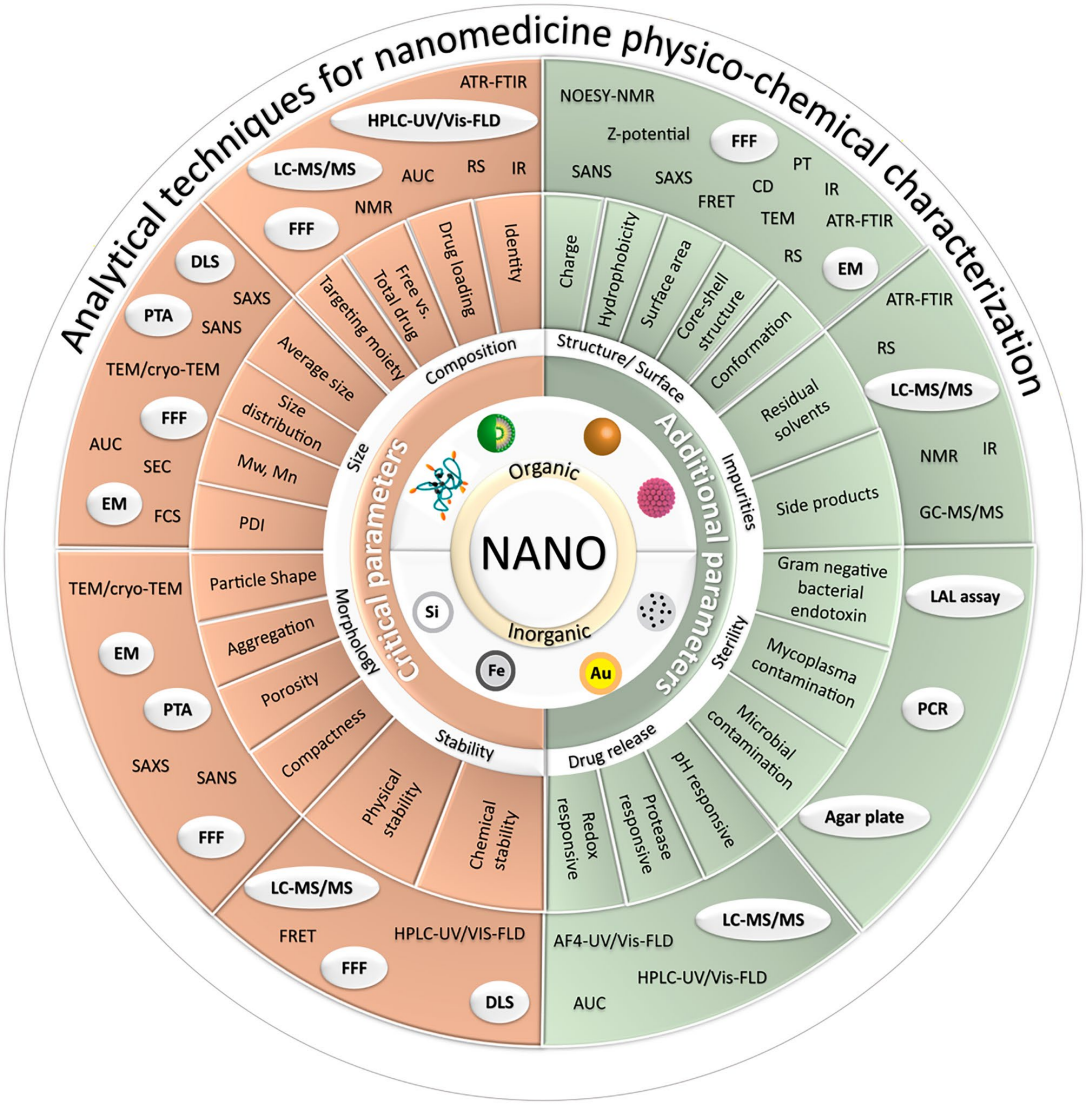
Critical and additional parameters of nanomedicines and corresponding analytical techniques for their characterization (NCL approved techniques are highlighted). LC–MS/MS liquid chromatography–mass spectrometry, FFF field flow fractionation, NMR nuclear magnetic resonance, AUC analytical ultracentrifugation, RS remote sensing, IR infrared, HPLC–UV high-performance liquid chromatography-ultraviolet, Vis-FLD visible-fluorescence detector, ATR-FTIR attenuated total reflection—Fourier transform infrared spectroscopy, NOESY-NMR nuclear Overhauser effect spectroscopy-nuclear magnetic resonance, SANS small angle neutron scattering, SAXS small angle x-ray scattering, FRET fluorescence resonance energy transfer, CD circular dichroism, PT potentiometry, TEM transmission electron microscopy, EM electron microscopy, GC–MS/MS gas chromatography–mass spectrometry, LAL assay limulus amebocyte lysate assay, PCR polymerase chain reaction, AF4-UV asymmetric field flow fractionation-ultraviolet, DLS dynamic light scattering, Cryo-TEM cryogenic transmission electron microscopy, FCS fluorescence correlation spectroscopy, SEC size-exclusion chromatography, PTA particle tracking analysis, Si silicon, Fe iron, Au gold

Standardized protocols and specific characterization strategies are of immense importance to the clinical translation of nanomedicines. Encouragingly, a nanomedicine-relevant roadmap has been issued by the NCI-NCL and NCL, who established a trans-disciplinary evaluation infrastructure covering a comprehensive set of pre-clinical nanomedicine characterization procedures (physical, chemical, in vitro, and in vivo biological testing). Figure 3 provides an overview of the analytical techniques used for the physicochemical characterization of nanomedicines, highlighting NCL-recommended techniques for the routine evaluation of corresponding parameters. For a detailed description of associated techniques, we direct the reader to previously published reviews from Gao et al. [122], Niño Pariente et al. [123], Coty et al. [124], and Melnyk et al. [100].

A comprehensive map of currently available standards regarding regulatory requirements for pre-clinical nanomedicine characterization has been recently detailed [37], and there exist a large number of critical overviews of existing and potential size evaluation standard methods [125–127] due to the enormous influence of particle size and size polydispersity in solution and biological fluids on the absorption, passage through biological barriers, biodistribution, and excretion of nanomedicines [128–130]. While dynamic light scattering (DLS), as the most common size determination method, provides accurate size determination of monodisperse samples, polydisperse samples generally require a multimodal approach that combines DLS batch analysis in a pre-screening step with high-resolution techniques such as field flow fractionation (FFF), particle tracking analysis (PTA), or transmission electron microscopy (TEM) in the following step.

The NCI-NCL and EUNCL have included FFF, PTA, and cryo-TEM analysis as part of the first-line characterization tools required for nanoparticle analysis, highlighting their importance to the nanomedicine field [125]. In addition, emerging techniques such as small-angle x-ray scattering (SAXS), analytical ultracentrifugation (AUC), differential scanning calorimetry (DSC), atomic force microscopy (AFM), and tunable resistive pulse sensing (TRPS) for size and morphology characterization all complement previously mentioned techniques.

Method sensitivity remains a crucial point in nanomedicine analysis, especially for those employed in determining free drug, TDL, stability testing, and release kinetics. The choice of detector, sample preparation, and method development represent crucial factors for these studies. The ICH has recognized the significance of method sensitivity and has proposed the “Q14 Analytical Procedure Development Guideline” [131] to harmonize scientific approaches and facilitate efficient analytical procedures.

The ICH quality guidelines provide an excellent starting point for the design of stability studies, thereby fostering more reliable pre-clinical results. Moreover, we believe that careful quality control of synthesized complex nanosystems with fit-for-purpose analytical methodologies will improve pre-clinical studies and the reproducibility of clinical efficacy studies.

### Stability

*Stability, degradability, and drug release studies* are mandatory to better understand biological responses due to their impact on safety. Nanomedicine stability represents an essential parameter from two aspects—(i) stability under storage conditions and (ii) systemic circulation stability, which refers to physical (stability against aggregation and agglomeration in cell media and plasma) and chemical stability (stability against chemical degradation in solution and after freeze/thaw cycles) over time. Unfortunately, the field suffers from a general lack of stability data even though drug delivery systems have demonstrated clinical relevancy [132].

From a *physical stability* standpoint, the ideal final nanomedicine product formulation maintains physical stability after incorporating an active moiety into a carrier. We note that the term stability refers to the full nanosystem *and* active payload, targeting ligand, drug linking moiety, and nanocarrier. Thus, stability studies should be performed for each nanomedicine decorated with a new moiety [100].

Nanomedicine instability can have two major consequences with clinical significance: (a) vehicle issues, in which particle agglomerations may produce an embolism, particularly within the pulmonary microvasculature following intravenous administration, and (b) activity issues, in which the homogeneous distribution of an active moiety becomes altered, affecting the nanomedicine’s therapeutic efficacy [65]. Factors that negatively affect vehicle stability under storage conditions or during synthesis (e.g., alterations in temperature, humidity, pH, oxygen concentration, light exposure, and addition of electrolytes) must be identified and controlled. The evaluation of a nanomedicine’s physical stability employs the determination of size, size distribution, morphology, and surface charge. Particle size stability in biological media affects safety and efficacy and should be considered a crucial quality parameter for nanomedicines. Recent FDA guidelines reported that plasma proteins influenced nanomaterial properties, including increased particle size due to the *protein corona* formation and particle aggregation or destabilization [116]. The detailed analysis of nanoparticles in complex biological media represents a challenge to low-resolution size determination methods such as batch DLS. Therefore, there exists an urgent need for methods that assess changes in physico-chemical parameters when a nanomedicine enters systemic circulation.

Asymmetric FFF (AF4) represents a promising method due to the gentle and sample-tailored separation inside the empty channel that maintains weak complexes such as nanoparticles and proteins. Affinity studies of nanomedicines with long circulation times to proteins by AF4 have been reported [125, 127, 133–137]. These examples, and existing AF4 standard operation procedures from the NCL [138], demonstrate that coupling AF4 to MALS or DLS represents a powerful and robust analytical technique for characterizing nanomedicines that may overcome limitations associated with batch mode light scattering techniques.

A recent focus on protein corona analysis may enhance our understanding of nanomedicine safety and efficacy. Techniques other than AF4, which include distinct microscopy techniques [139], have been combined to provide a more detailed picture of the protein corona’s biological effects [140, 141]. The protein corona can affect nanoparticle biodistribution by negatively impacting targeting and obscuring imaging abilities in cases of diagnostic/theranostic approaches [142]. Interestingly, analysis of interactions between nanoparticles and blood plasma has determined that a nanoparticle undergoes a range of distinct interactions with plasma components [141]. This funding suggests that an administered nanomedicine may suffer multiple fates, which may affect clinic approval. Engineering and/or detailed analysis of the protein corona can also improve parameters affecting therapeutic outcomes, including biocompatibility, toxicity, targeting, payload capacity, and disease detection [140]. Studies have provided evidence supporting early disease detection through protein corona molecular fingerprinting and the improvement of blood proteomic analysis and biomarker discovery through protein corona analysis. Studies of anti-fungal agent amphotericin B-containing liposomes (AmBisome®) resulted in the discovery of over sixty potential biomarker proteins that differentiated non-infectious acute systemic inflammation from sepsis [143]. Notably, the exact structure of the protein corona remains a controversial topic due to variability and fluctuations over time in vitro and rapid exchange in vivo [33]; therefore, the full impact of the protein corona and the possibility of extrapolating in vitro evaluations in vivo remains contentious.

Plasma stability and drug release kinetic studies require the quantification of free drug *versus* the drug existing in nanomedicine due to the greater inherent complexity than small drugs or biologics [25, 144, 145]. To note, progression to biological characterization requires a free drug content assessment [100]. Ph. Eur and USP requirements for APIs are highly specific concerning the concentration range for a particular compound, both for the active ingredient and the dosage form. Each monograph details specific *chemical stability* requirements, whereby the methods applied must be stability-indicating (i.e., differentiate between parent drug and degradation products) and ensure the presence of a fixed amount of API during the manufacturer-assigned shelf-life.

When targeting diseased tissues, a nanosystem should display stability in systemic circulation to enhance the delivery of the associated bioactive agent to the required site of action. Therefore, the *free drug content* has less significance to efficacy but more significance to off-target exposure-toxicity relationships. The presence of the free drug in blood suggests nanomedicine instability and incomplete targeting (when the blood is not the desired target). Low plasma chemical stability leads to drug release in the bloodstream due to the degradation of the nanocarrier or existing bonds between the drug and a carrier; however, targeted drug release at the required site of action in response to a specific trigger (endogenous or exogenous) represents a fundamental factor in the success of nanomedicine-based therapies. Both plasma stability studies and drug release kinetic studies must verify drug delivery as a nanomedicine component and not the codelivery of the drug alongside the nanocarrier (which could be considered an important impurity). In liposomal prodrugs, regulatory guidance has recommended developing bioanalytical methods to distinguish free and encapsulated APIs [146]. The direct measurement of a released API can present a significant technical challenge when present at low concentrations alongside a large excess of encapsulated drug non-covalently associated with a given nanocarrier (i.e., rapid clearance drugs); however, strategies that model PK profiles for encapsulated and released drug may be utilized [147].

The utility and limitations of modeling remain controversial. An incomplete understanding of absorption, distribution, metabolism, and excretion (ADME)-specific mechanisms have limited modeling efforts, as evidenced in a study of how macrophages affect stable liposomes [148]. Notably, nanomedicine clearance from the circulation by the mononuclear phagocyte system (MPS) [149] depends on both size and shape [149] and directly influences PK and PD [150, 151]. Overall, modeling and the prediction of effective doses early in development remains challenging and requires further development.

Biosensitive drug-release requires analyses in scenarios ranging from simple mixtures of enzymes, cell media, or pH buffers to biological fluids such as plasma, cerebrospinal fluid, and urine. Such analyses can identify metabolites deriving from nanomedicine degradation during in vivo studies and determine dose schedules and suitable administration routes, which represent essential elements for efficient delivery to required sites of action [152, 153]. A first step in separating free *versus* bound drug fractions typically employs ultrafiltration with a molecular weight cut-off centrifugal filter, solid-phase extraction, size exclusion, AF4, and liquid chromatography [154–160]. After separation, the NCL recommends LC–MS/MS to determine free drug concentration referenced against quantification standards in the European pharmacopeia monographs (in the case of non-availability, member state monographs) specific to the individual drug. Alternatives to MS for coupling with HPLC include UV–Vis, fluorescence, and charge aerosol detectors. Recent research has highlighted analytical ultracentrifugation as a rapid and straightforward method for separating and determining free and bound drugs using a single measurement to improve total analysis time and reduce experimental complexity [161]. The stable isotope tracer ultrafiltration assay (SITUA) represents a recently developed drug release method for assessing nanomedicine stability in human plasma[145, 162]. A stable isotopically labeled drug (D*) added to nanomedicine-containing plasma equilibrates with plasma proteins similarly to the unlabeled normoisotopic drug released from the nanomedicine. Thus, the %D* bound to plasma proteins provides a reliable estimation of unencapsulated drug fractions, which, in turn, can be used to determine nanomedicine drug release in biological matrices.

Finally, drug release and free drug fraction quantification represent critical parameters for *bioequivalence* studies required to evaluate therapeutic equivalence between two drug products and, for example, support biowaiver requests for lower doses [25, 163]; however, investigating the bioequivalence of nanomedicines and other non-biological complex drugs remains a challenging task [25, 164].

### Pre-clinical pharmacology and toxicology studies

The nanomedicine complexity has confined them to basic research, with a poor understanding of their biological effects and interactions in the body hindering clinical translation. Pre-clinical pharmacology and toxicology represent essential elements in facilitating translation. Drug discovery remains a complex process and involves continuous iterations to optimize the pharmacological and drug-like properties of a candidate and minimize potential side effects and toxicities.

A lack of specific and systematic bioanalysis protocols for development and characterization and the absence of specific regulatory frameworks for nanomedicine have also hindered progress. The importance and significance of the physicochemical characterization of nanoparticles are well documented; these steps aim to correlate a nanoparticle’s physicochemical characteristics to biocompatibility, biodistribution, toxicity, bioaccumulation, and clearance [165–167].

Research organizations such as the NCL have assembled an extensive database derived from their wide-ranging collaborations that may guide nanomedicine development and potential ADME/safety issues [115]. Data sharing and an increased level of understanding may contribute to developing robust guidance documents for regulatory purposes. For example, the NCL currently contributes industrial guidelines to aid the development of nanomedicines such as liposomes, colloidal metal nanoparticles, and polymeric nanoparticles, thereby addressing questions raised by the US FDA. Since 2004, the NCL has characterized more than 300 products, with seven entering clinical trials [168]. More importantly, NCL has provided more than fifty standardized analytical cascade protocols to evaluate the pre-clinical toxicology, pharmacology, and efficacy of nanoparticles and devices [169, 170]. As a whole, these studies provide an in-depth understanding of the molecular pathways related to pharmacological output and contribute to further clinical progression [100]. Also, questionnaires from the NCL directed to distinct groups of regulatory scientists aim to ensure the relevance of developed/validated methods for regulatory purposes and that obtained information supports regulatory decision-making [171].

Additional obstacles that have hindered progress in nanomedicine translation include the following:

- (i) elevated nanoparticle complexity, which provides a challenge to the definition of CQAs
- (ii) a lack of structure-activity relationships (SARs), which provides a challenge to the prediction of biological outcomes
- (iii) misleading results from stability and drug release studies in standard buffers. For example, in vitro assays measuring drug release/stability of nanomedicines in plasma can better predict in vivo PK parameters.
- (iv) a lack of relevant controls and benchmark studies. For example, comparisons with standard care/gold-standard treatments, empty platforms, free APIs, non-targeted formulations, and comparisons at equitoxic and equal doses.
- (v) a lack of predictive in vitro and in vivo models, inappropriate animal numbers, and a lack of in vitro/in vivo correlations.

Pre-clinical studies of nanomedicines should also include randomization and blinding to reduce bias. Common causes for early clinical failure of nanoformulated drugs include endotoxin contamination, the induction of cytokine storm, hypersensitivity reactions, complement activation, thrombogenicity, and API immunotoxicity. Most toxicities can be rapidly assessed through available in vitro models, many with well-established in vitro-in vivo correlations [172].

In vitro* screening studies* can identify biocompatible candidates, improve our understanding of nanomedicine-cell interaction, and contribute to optimization before formal toxicology assessments in vivo. Although extremely useful at a first stage screening, in vitro assessments do not fully replicate the in vivo scenario; however, they can reduce risk factors during nanomedicine development. Assessments include evaluations of therapeutic activity and mechanism of action, cellular uptake, toxicity (necrosis, apoptosis, oxidative stress, and autophagy), and immune responses (blood contact properties and cell-based assays) [168]. At the cellular level, surrogate biological in vitro systems such as human or other mammalian-derived cell cultures offer an easy and accessible means to evaluate nanomedicines [173]; however, traditional two-dimensional (2D) models often fail to recapitulate the complexity of in vivo biological systems. Indeed, the lack of heterogeneous cell populations (including immune cells), extracellular matrix and serum proteins, and dynamic cell-to-cell interactions hamper nanomedicine evaluation in traditional 2D cell culture. Furthermore, poor distribution and the inability to compensate stress via homeostatic balances can foster overestimated drug toxicity predictions and limit the understanding of a given nanomedicine’s behavior in vivo. Consequently, discontinuations of pre-clinical studies have occurred in the early phases of nanomedicine development [174, 175]. At an intermediate stage, newly developed biomimetic devices may accurately model nanomedicines' behavior in vivo. Advances include bioprinting [176, 177] and organ/tumor-on-a-chip models may support the accurate recapitulation of the interplay of nanomedicines with physiological barriers [178–180], while three-dimensional spheroid/organoid cultures in microfluidic devices may reveal how interstitial flow affects cell binding and how particle size influences nanoparticle diffusion and accumulation [181–183]. In cancer research, the development of organotypic multicellular tumor spheroids aims to preserve and faithfully reproduce tumor structure by involving stromal and immune cell components. Furthermore, tumor‐derived spheroids (TDS) and multicellular tumor spheroids (MCTS) can maintain a similar level of cell clonality and metabolic activity as tumors in vivo. The rapid and affordable nature of TDS and MCTS and their in vivo-like gene expression profiles (including therapeutic resistance-associated genes) support their implementation as suitable model systems for the investigation of nanomedicine penetration, accumulation, and cell internalization, as well as drug efficacy and prediction of drug resistance [184–186].

Of note, research infrastructures such as EU-OpenScreen support academia-industry collaboration towards developing technologies in the field of high-throughput screening using complex cellular models to facilitate, accelerate, and enhance early-phase drug discovery and technological development [177].

Pre-clinical safety evaluations using NCL standardized assay protocols to generate data on physicochemical characterization and bio-interactions for hazard assessment have been reported in an effort to refine the methodology required to define SARs in terms of nanomedicine safety and efficacy [33, 187]. Validation of these approaches requires well-characterized reference materials and their implementation by researchers and regulatory authorities. Quantitative structure–activity relationships (QSARs), which are employed to correlate physicochemical properties and cytotoxic effects, can identify non-qualifying materials and minimize the number of in vitro experiments required, hence avoiding costly in vivo testing and mitigating the risk of off-target effects. While described machine learning models can predict nanotoxicological outcomes, these approaches only model specific types of nanomedicines, extracting data from the literature and subsequently testing associated strategies in various in vitro systems and cell line models [187]. Perturbation models or quasi-QSARs may build more accurate biological models as they capture the impact of exposure conditions and other experimental parameters. The construction of databases and multisource data extraction remains an ongoing task and a research priority. Undoubtedly, this knowledge will guide nanomaterial design according to safe-by-design principles (as presented by the NANoREG and ProSafe European initiatives) to assist regulatory agencies and industrial concerns. An extension of this approach to diverse nanoparticle platforms and biological responses could facilitate a deeper understanding and better control of the nano-bio interface and support the rational design of safe, effective, and patient-specific nanomedicines [93].

Problems facing in vivo* assessments* include significant discrepancies between pre-clinical and clinical data. The lack of predictability regarding the benefit of a given nanomedicine to a patient may derive from dependence on PK efficacy, tissue distribution, target site accumulation, penetration, and drug release at the target site. These aspects impact in vivo performance and differ between animal models and patients, thereby underscoring a lack of disease models that faithfully recapitulate human disease [188–190]. The inconsistency of the enhanced permeability and retention effect (EPR) in primary tumors and metastases provides an important barrier to nanomedicine targeting and penetration [191]. Moreover, tissue morphology, stroma, and macrophage population can vary inside a tumor, across tumors in a patient, and among different patients [192]. We currently lack an understanding of the differential influence of the EPR effect in these scenarios and the impact on the lymphatic system. Thus, success in translation (not unique to nanomedicines) relies on the development of animal models that mimic the heterogeneity and anatomical histology of human diseases. Relevant approaches include the development of patient-derived xenografts (PDXs), humanized mouse models [193], and genetically engineered mouse models (GEMMs) with aggressive metastasis [194]; however, the development and application of these models remain time-consuming and expensive.

Alternative biological models for nanomedicine high-throughput screening include model animals such as nematodes (*Caenorhabditis elegans*), frogs (Xenopus laevis), chicken embryos, zebrafish (*Danio rerio*), and rodents (*Mus musculus*) [195–198]. Zebrafish recapitulate many human biological and genetic features while offering high-resolution (fluorescence) imaging and the straightforward, low-cost evaluation of in vivo drug stability and functionality and nanomedicine biodistribution. For example, the transparent, fully developed adult zebrafish line known as *casper* allows monitoring of fluorescent reporter expression and the attainment of imaging data regarding nanomedicine behavior [199]. As in humans, the intravenous administration of nanomedicine into zebrafish activates the plasma proteome (which includes apolipoproteins and complement factors) that induces opsonization of nanomedicines in the circulation, thereby allowing the evaluation of drug stability. Overall, zebrafish larvae are largely preferable over rodent models to investigate nanomedicine toxicity, biodistribution, and stability [197]. Recent research has also reported that zebrafish larvae recapitulate human infectious diseases (e.g., tuberculosis and granulomas) with more accuracy than rodents [200, 201]; however, the limited size of tumor xenografts in zebrafish has raised concerns regarding the conservation of tumor cellular hierarchy (hypoxic/necrotic core *versus* proliferative area) and the tumor microenvironment in cancer-based studies [202]. At the genomic level, rodents share 84% of human genes as compared to 76% and 80% in zebrafish and chicken, respectively [202]; however, rodent models are more expensive, time-consuming, and suffer from restrictions to imaging, which limits the number of nanomedicines assessed in a single study. Finally, despite a focus on collecting data on post-treatment survival and tumor size (in the case of solid tumors), the mechanism of action of evaluated nanomedicines should be explored by assessing appropriate endpoints and referring the results to a proper control [203, 204]

Evaluating a given nanomedicine in multiple pre-clinical models with different tumor characteristics can foster a deeper understanding of the full impact of tumor structure on nanomedicine efficacy. These studies require evaluating nanomedicine administration in at least two relevant animal models to provide reproducible results for a specific disease and not the animal employed, thus allowing the extrapolation of dosing values and scheduling parameters to clinical trials. Notably, animal models that reflect only some clinical disease aspects can provide valuable data that predict applicability to a specific patient sub-group [205]. While small molecule phase I dose-escalation studies in human patients evaluate three to five doses, this can rise to as high as fourteen for nanomedicines, which may derive from the initial analysis of starting doses in canine models that can present hypersensitivity to nanomedicines [147]. Overall, researchers should interact with regulatory bodies at early stages to compile and organize relevant information for submission to the FDA while filing an investigational new drug (IND) application for nanomedicines requesting authorization for clinical trials.

The development of *biomarkers*, *imaging studies,* and *companion diagnostics* addressing PD and trafficking can identify optimal nanomedicine candidates and potential responders to reduce failure rates in late-stage clinical development. Indeed, a growing body of data emerging from industry-sponsored clinical studies may ultimately enable patient selection strategies and strengthen the mechanistic underpinnings of nanomedicines [206]. For example, studies that visualized radioisotopes within liposomal drug products by PET or SPECT imaging after administration to patients with solid tumors established particle accumulation and retention at tumor sites [207, 208]. Similarly, MRI imaging of patients following ferumoxytol administration, a 30-nm iron oxide particle with contrast properties, indicated particle accumulation in tumors and substantial variability in tumor uptake [209]. In addition, preliminary data from a small number of patients suggested that the uptake of ferumoxytol or ^64^Cu-labeled HER2-targeted liposomes in tumors may correlate with intratumoral drug concentration and treatment response measurements. Similarly, prostate-specific membrane antigen (PSMA) expression data in archival tissue specimens from solid tumor patients that attained clinical benefit from BIND-014 treatment (a docetaxel-loaded PSMA-targeted PEG-polylactic acid nanoparticle) suggested a correlation between PSMA expression and therapeutic response [210]. Overall, biomarkers can strongly impact the clinical success of nanomedicines by helping to stratify patient cohorts [211]. For example, the use of biomarkers for patient stratification has contributed to the successful clinical development and approval of four antibody–drug conjugates; however, a lack of biomarkers has been noted as the reason behind the failure of cancer nanomedicines based on liposomes, polymeric nanoparticles, and micelles (including CRLX101 [camptothecin loaded PEG-cyclodextrin nanoparticles], or NK105 [paclitaxel-loaded PEG-polyaspartate-based micelles]).

Regulatory authorities view nanomedicines on a case-by-case basis when gathering safety data. *Perspective articles* provide guidelines on the development of specific nanoparticle-based drug delivery systems [118]. Only around 5% of initially evaluated entities (including INDs, nanomedicines, and non-nanomedicine-based therapeutics) lead to the submission of a New Drug Application (NDA) and market authorization [146]. The strategies employed by regulatory authorities to evaluate nanomedicine *safety/toxicity* and compatibility are often adapted from “conventional” medicinal products [64, 149, 192, 212, 213]. From a regulator's perspective, the API of a nanomedicine dictates the specifications analyzed within the regulatory context; however, the multicomponent nature of nanomedicines raises toxicity concerns.

As the biodistribution of nanomedicines possesses a different profile from the parental API, uptake in specific organs may promote local overexposure. Furthermore, beyond the intrinsic toxicity of the bioactive agent and the nanomedicine as a whole drug product, the multiple components may also induce unexpected toxicities, e.g., excipients lacking adequate testing in humans. Therefore, all components, including the drug-free nanocarrier and the whole construct, must be considered in preclinical PK/PD studies at different doses if they have not been previously approved.

Regulatory agencies consider preclinical toxicity tests for small-molecule drugs useful for nanomedicines when conducted in at least two animal models, over extended treatment periods, and multiple doses [214]. The battery of tests includes acute and repeat-dose studies, safety pharmacology, genotoxicity, developmental toxicity, immunotoxicity, and carcinogenicity, typically employing two animal species (usually rat and dogs).

Challenges for the evaluations of ADME/toxicity in nanomedicine development include interactions with the immune and/or hematological systems [206, 215–217]. Endotoxin contamination interferes with the detection of nanomedicine-induced toxicity by inducing a non-specific immune response [213, 218]. The complement activation cascade plays a crucial role in immunological side effects, and nanomedicine-blood cell interactions may contribute. Therefore, understanding how nanomedicines interact with coagulation factors, as complement activation can be dose-limiting, remains an essential task, while evaluations of organ function, phagocyte activation, oxidative burst, cytokine release, hemolysis, thrombogenicity, effects related to protein corona, and antigenicity can inform on nanomedicine toxicity [213].

The NCL has suggested in vivo-in vitro correlation (IVIVC) methods to determine acute toxicities by hemolysis, complement activation, pyrogenicity, cytokine induction, and MPS uptake; however, the determination of thrombogenicity, myelosuppression, immunosuppression, and hypersensitivity is more complex compared with small drugs due to the differential PK/PD and biodistribution of nanomedicines given the absence of reliable models. Therefore, each distinct nanomedicine requires specific studies. Protein corona analysis may provide an adequate indicator of nanomedicine’s stealth properties but cannot accurately predict toxicity; therefore, specialized immunotoxicity tests must be performed. The use of in silico modeling for the prediction of nanomedicine-induced immunogenicity has been proposed as a “personalized safety” approach. With few exceptions, animal cells or models do not entirely predict human immune reactivity, and patient-derived xenografts in immunodeficient mice usually lack relevance regarding immunogenic evaluations [219]. The preferred implementation of immunocompetent models may accelerate clinical translatability given the affinity of nanosized materials for immune cells [220].

Recently, the immunology team at the NCL published seven protocols for the evaluation of immunotoxicology aspects of nanomedicines [221], including complement activation and oxidative stress in T lymphocytes, antigen presentation and stimulation, and the detection of naturally occurring antibodies to PEG. For example, variations in shape, size, and composition of nucleic acid nanoparticles induce distinct immunostimulatory profiles [221]. Recent studies have described how the nanosized carrier employed for delivery provides an additional means to tailor nanomedicine immunorecognition (e.g., lipid-based platform *versus* dendrimers display differences in cytokine induction) [222].

For biological entities such as proteins, peptides, or antibodies, an innovative product must follow the regulations defined for biological medicinal products and NCEs [13]. Regulatory guidance documents for the non-clinical evaluation of anticancer agents, such as the ICH S9 guideline [132], represent the starting point. These guidelines include toxicological evaluation in rodent and non-rodent species but recommend a limited evaluation of the parent drug and carrier. Concerning non-rodent species, several canine studies have revealed unusual sensitivities to nanoparticles or components such as polysorbate surfactants [223–225]. Due to these findings, phase I studies of Abraxane and BIND-014 employed toxicology studies in non-human primates, while initial studies of the parent compounds (paclitaxel and docetaxel, respectively) employed canine models.

The clinical underrepresentation of imaging agents [226], among the earliest nanomedicines in clinical use, derives from a lack of selective-targeting ligands and receptors, difficulties in synthesis and scale-up, and drawbacks related to biocompatibility and regulatory demands. Given the likely administration of diagnostics to a healthy population and the associated unacceptable safety risks, safety assessments should occur early in preclinical development and focus on toxicities in target organs (i.e., renal, respiratory, and cardiovascular systems). The elevated aggregation tendency of nanomedicines with increasing concentration represents an additional concern for imaging agents administered at high mg/kg doses and is a particular concern for computed tomography.

In general, the formulation of conventional drugs into nanomedicines provides improved safety and biocompatibility, and reduced toxicity; however, nanomedicines such as carbon nanotubes or quantum dots entail additional concerns. Potential toxicity and deleterious immunological effects observed in preclinical/clinical studies may compromise their future translation [13].

## Conclusions

Despite the promising advances made in preclinical animal models, the clinical translation of nanomedicines remains a slow, biased, and often failed affair. There exists a general lack of specific protocols, and the characterization of materials and biological mechanisms and the statistical analyses often employed remain inadequate. Moreover, the vast and significant heterogeneity of models adopted, a reluctance to share results, and the inaccuracy of study design have hampered the translation of nanomedicines into late clinical trial stages [227]. As a result, only 20–25% of the 67 preclinical studies related to general biology have been translated to oncology. In contrast, inconsistent results observed among published and industry-obtained data [227, 228] and the inability to find appropriate commercial partners due to the challenging gaps in translation have prompted the termination of the remaining studies. In cancer, the success rate of 94% of studies in phase I clinical trials drops to 48% in phase II and 14% in phase III [229]. The clinical application of nanomedicine strongly relies on the intensive and meticulous characterization of associated properties, as minor changes in chemistry or manufacturing processes can result in significant alterations to biodistribution and tolerability. Failures during clinical translation may be mitigated by defining stringent criteria (such as tests and quality control checks) during nanomedicine design and development. Firstly, biocompatibility and immunotoxicity should be evaluated through the characterization of nanomedicine pharmacotoxicology, which includes defining the therapeutic index, dosage regimen, maximum tolerated dose, route, and target of drug administration. Drug hemolysis, complement activation, cytokine release, opsonization, phagocytosis, and PK (ADME) studies represent just part of the in vitro and in vivo data correlations required for the clinical translation of nanomedicines.

Multidisciplinary teams developing nanomedicines by bridging material science, new technology platform characterization, disease models better resembling the targeted clinical conditions, while shaping the current regulatory frameworks to science-based standards, will certainly generate the data required to grant marketing authorization of disruptive technologies demanded to tackle world’s unmet needs for health care and treatments.

## Abbreviations

2D: Two-dimensional
3D: Three-dimensional
ADME: Absorption, distribution, metabolism, excretion
AF4: Asymmetric field flow fractionation
AFM: Atomic force microscopy
API: Active pharmaceutical ingredient
ASTM: American Society for Testing and Materials
ATR: Attenuated total reflection
Au: Gold
AUC: Analytical ultracentrifugation
CD: Circular dichroism
CMA: Critical manufacturing attributes
COVID-19: Novel coronavirus disease 2019
CPP: Critical process parameter
CQA: Critical quality attribute
cryo-TEM: Cryogenic transmission electron microscopy
CVD: Chemical vapor deposition
D: Unlabeled normisotopic drug
D*: Stable isotopically labeled drug
DLS: Dynamic light scattering
DoE: Design of Experiments
DSC: Differential scanning calorimetry
EC: European Commission
ECHA: European Chemical Agency
EFSA: European Food Safety Authority
EM: Electromagnetic waves
EMA: European Medicines Agency
EMU: European Medicines Union
EPR: Enhanced permeability and retention effect
ETPN: European Technology Platform on Nanomedicine
EU: European Union
NCL: Nanomedicine Characterization Laboratory
FCS: Fluorescence correlation spectroscopy
FDA: US Food and Drug Administration
Fe: Iron
FFF: Field flow fractionation
FLD: Fluorescence detector
FRET: Fluorescence resonance energy transfer
FTIR: Fourier transform infrared spectroscopy
GC–MS: Gas chromatography-mass spectrometry
GEMM: Genetically engineered mouse model
GMP: Good manufacturing practice
HPH: High‐pressure homogenizers
HPLC: High-performance liquid chromatography
HPLC–UV: High-performance liquid chromatography – ultraviolet
ICH: International Council of Harmonization
IND: Investigational new drug
IPRP: International Pharmaceutical Regulators Program
IR: Infrared
ISO: International Organization for Standardization
IVIVC: In vivo-in vitro correlation
LAL: Limulus amebocyte lysate
LC–MS/MS: Liquid chromatography with tandem mass spectrometry
MCTS: Multicellular tumor spheroids
MHLW: Ministry of Health, Labor and Welfare
MHRA: Medicines and Healthcare Products Regulatory Authority
MPS: Mononuclear phagocyte system
MS: Mass spectrometry
NBCD: Non-biological complex drug
NCL-NCI: Nanotechnology Characterization Laboratory of the National Cancer Institute
NDA: New Drug Application
NMR: Nuclear magnetic resonance
NNI: National Nanotechnology Initiative
NOESY: Nuclear overhauser effect spectroscopy
OECD: Organization for Economic Co-operation and Development
PAT: Process analytical technology
PCR: Polymerase chain reaction
PD: Pharmacodynamics
PDX: Patient-derived xenograft
PEG: Polyethylene glycol
Pharm Eur: European Pharmacopoeia
PK: Pharmacokinetics
PKPD: Pharmacokinetic-pharmacodynamic
PLGA: Poly(lactic-co-glycolic acid)
PLM: Product life-cycle management
Ppm: Parts-per-million
PRINT: Particle replication in non-wetting templates
PTA: Particle tracking analysis
QbD: Quality-by-Design
QTPP: Quality target product profile
RCS: Risk control strategy
REACH EC: Registration, Evaluation, Authorization, and Restriction of Chemicals European Chemical Legislation
REFINE: Regulatory Science Framework for Nano(bio)material-based Medical Products and Devices
RS: Remote sensing
SANS: Small-angle neutron scattering
SAR: Structure–activity relationship
SARS-CoV-2: Severe acute respiratory syndrome coronavirus 2
SAXS: Small-angle x-ray scattering
SEC: Size-exclusion chromatography
Si: Silicon
siRNA: Short-interfering RNA
SITUA: Stable isotope tracer ultrafiltration assay
SMEs: Small and medium-sized enterprises
TDL: Total drug loading
TEM: Transmission electron microscopy
TRPS: Tunable resistive pulse sensing
TTR: Transthyretin
UK: United Kingdom
USA: United States of America
USD: United States Dollar
USP: US Pharmacopoeia
UV–Vis: Ultraviolet and visible absorption spectroscopy
